# Census tract based income level and lipid levels in urban pediatric primary care: a retrospective study

**DOI:** 10.1186/s12887-016-0622-x

**Published:** 2016-07-08

**Authors:** Enid E. Martinez, Peter W. Forbes, Sharon E. O’Brien, Sarah D. de Ferranti

**Affiliations:** Department of Medicine, Boston Children’s Hospital, Harvard Medical School, Boston, MA USA; Department of Pediatrics, Boston Medical Center, Boston, MA USA; Division of Critical Care Medicine, Department of Anesthesiology, Perioperative and Pain Medicine, Boston Children’s Hospital, Harvard Medical School, Bader 634, 300 Longwood Avenue, Boston, MA 02115 USA; Clinical Research Program, Boston Children’s Hospital, Harvard University, Boston, MA USA; Department of Cardiology, Boston Children’s Hospital, Harvard Medical School, Boston, MA USA

**Keywords:** Socioeconomic status, Cholesterol, Childhood, Obesity

## Abstract

**Background:**

Lower socioeconomic status has been associated with adverse lipid levels in adult populations. Childhood dyslipidemia is a risk factor for future cardiovascular disease. However, studies examining relationships between socioeconomic indicators and lipid levels in children are limited. To examine the relationship between income level and lipid levels in childhood.

**Methods:**

We conducted a retrospective chart review of primary care patients, ages 2 to 18 years, who had lipid levels drawn at two large pediatric practices in Boston, MA between August 01, 2008 and August 31, 2010. Income level was determined using geocoding census tract data. Analysis was performed using t-test, Anova and Spearman correlation coefficients. BMI percentile, age, sex, race/ethnicity, and site were adjusted for on multivariate analyses.

**Results:**

Reviewing 930 charts of patients with measured lipid levels, 730 had a valid address, no previously diagnosed lipid disorder and met other study eligibility criteria. Mean total cholesterol level did not vary by income level (low 155.5 mg/dl ±26.9, moderate 153.5 mg/dl ±30.4, middle 155.3 mg/dl ±26.6 and high income 155.5 mg/dl ±27.9; *p* = .87) on multivariate analysis. Income level was not related to LDL, HDL, or triglycerides.

**Conclusions:**

In this analysis of children cared for in two urban pediatric primary practices, there was no association between income level determined by census tract and lipid levels in childhood. If confirmed in prospective investigations in other geographical locations, income level may not be a key driver of childhood lipid levels.

## Background

Dyslipidemia, a cardiovascular risk factor, is prevalent in the pediatric population [1–4]. Dyslipidemia is found in 8 and 15 % of child and adolescent populations, respectively [3, 5–7]. Childhood dysipidemia may persist into adulthood and has been associated with future cardiovascular disease [8–10].

Socioeconomic indicators influence accessibility to resources that can affect behaviors, such as diet and activity, relevant to the development of dyslipidemia, among other cardiovascular disease risk factors. In adult populations socioeconomic indicators such as education and income level have been shown to impact cardiovascular disease risk factors, including lipid levels [11, 12]. In pediatric populations, some studies have identified an inverse relationship between socioeconomic indicators, such as household income, and dyslipidemias, whereas other found no relationships [13–15]. These studies have included large patient cohorts but have focused on the adolescent subcohort, and some have lacked diversity in ethnicity and race [14–18]. Socioeconomic indicators in childhood have been found to track into adulthood [19].

In this study we investigated whether there was an association between income level and dyslipidemia in a pediatric multiethnic cohort of 2 to 18 year olds. We performed a retrospective study to examine the relationship between lipid levels, total cholesterol (TC), high-density lipoprotein (HDL), low-density lipoprotein (LDL) and triglycerides (TG), and income level using census tract geocoding data in patients from two urban pediatric primary practices. Delineating the relationship between socioeconomic indicators and dyslipidemias may contribute to the understanding of the mechanisms underlying socioeconomic contributions to cardiovascular risk factors and aid in the development of effective intervention strategies.

## Methods

Subjects were included in this retrospective chart review if they were 2 to 18 years of age, attended a primary care visit at either of two large urban primary pediatric care centers in Boston, Boston Medical Center (BMC) or Boston Children’s Hospital (BCH), between August 01, 2008 to August 31, 2010, and had a recorded first-time TC lab value. Patients were excluded if their electronic medical records (EMR) lacked a complete street address, as this would not allow for collection of census tract data. In addition, patients were excluded if they had a diagnosis of hyperlipidemia or an identifiable secondary cause for hyperlipidemia prior to the study period. Patients with a prior diagnosis of hyperlipidemia or secondary cause for hyperlipidemia were often followed in subspecialty clinics, endocrinology and/ or cardiology, and therefore were likely to be receiving dietary or physical activity counseling and/or lipid lowering medications.

Potentially eligible patients were identified by an automated screen of the EMR. Patients were considered for inclusion if they completed primary care visits between August 1, 2008 and August 31, 2010. August 01, 2008 was chosen as the earliest date for identifying eligible patients because it was one month after the publishing of the updated America Academy of Pediatrics (AAP) lipid screening guidelines [20]. Potentially eligible patient charts were reviewed by the primary author for all inclusion and exclusion criteria. Once eligibility was confirmed, the following additional data were retrieved when available: sex, height, weight, body mass index (BMI) percentile based on the Centers for Disease Control and Prevention (CDC) standard growth curves, past medical history, medications, family history and smoking history and other lipid data including TG, LDL and HDL levels [21, 22]. Because this was a retrospective chart review, fasting state could not be verified. Patterns in lipid levels by race/ethnicity have been described [6, 18]. Self-identified ethnicity/race was recorded because lipid levels are known to vary by race ethnicity.

Lipid levels were drawn and processed per institutionally approved guidelines for clinically indicated diagnostic testing. The indication to draw a comprehensive lipid panel versus total cholesterol per patient was guided by the lipid screening guidelines published by the AAP in 2008 as interpreted and implemented by individual physicians in the two separate pediatric primary care clinics [20]. Per the study’s inclusion criteria, all subjects had a TC value; the availability of HDL, LDL and/or triglyceride serum levels was dependent on the individual physician’s discretion. The serum LDL values were measured directly if requested by the primary care physician and otherwise calculated using the following equation [LDL = total cholesterol – HDL – (triglycerides/5)] when HDL and triglyceride values were available in addition to the total cholesterol.

Income level was determined based on FFIEC geocoding census tract data, (http://www.ffiec.gov/Geocode/default.aspx) from the year of the clinic visit and the patients’ recorded street address [23]. Income levels - low, moderate, middle and high - are determined by the FFIEC comparing the median family income in the tract to national median family income [24]. A census tract is usually a contiguous area covering 1,200 to 8,000 people established either locally or by the Census Bureau, which largely remains unchanged over time. (https://www.census.gov/geo/reference/gtc/gtc_ct.html) Census tract data in this study were based on 2000 Census questionnaires, which provide household and population level social and economic status information. Census tract data have been shown to correlate with individual socioeconomic indicators and have been used as proxy measures of household socioeconomic indicators when individual patient data are not available [25–27]. Study data were collected and managed using REDCap electronic data capture tools hosted at BCH [28]. Individual applications to the IRB for the Boston Medical Center and Boston Children’s Hospital were submitted and both institutions provided an IRB exempt status for this retrospective study.

Our primary analysis examined the relationship between TC levels and income level determined by census tract data. Secondary analyses determined associations between other lipid levels including HDL, LDL and TG and income level. Power calculations performed at time of study design were based on Resnicow et al., which demonstrated a 6 mg/dl ± 29 mg/dl difference in TC between subjects from low socioeconomic status (SES) schools versus mid/high SES school [17]. A sample size of 734 patients was determined to identify a significant difference in TC based on income level category. Chi-squared tests and Fisher’s exact tests were used to evaluate categorical data and unpaired (two-sample) t-tests were used to evaluate continuous variables. All means were reported as raw means. Since TG values were skewed, this measure was log-transformed before analysis; *P*-values for TG are from analysis of log-transformed data. Multivariable linear regression was used to test for association between lipids and income level adjusting for the following covariates: BMI percentile, age, sex, race/ethnicity (four categories) and site (BCH vs. BMC). SAS software (version 9.2, Cary, NC) was used for all computations and *p* < .05 was the criterion for statistical significance for all tests. Data were reviewed and double-entered and analyzed between July 2011 and 2012.

## Results

A total of 923 charts were reviewed, of which 730 met inclusion criteria (362 from BCH and 368 from BMC). Specifically 49 subjects were excluded due to a documented previous diagnosis of hyperlipidemia. While TC was available for all patients by design, HDL, and TG levels were identified in 611 and 402 patients respectively. LDL was directly measured in 330 patients and calculated in 81 patients whom had available an HDL and TG value in addition to TC, resulting in a total of 411 patients included in the LDL analysis. Thirty-two percent of the study cohort was obese by CDC BMI percentile criteria, ≥95th percentile. Patient characteristics were described by income level based in Table 1.

**Table 1:** Characteristics of patients by FFIEC geocoding designated income level

Characteristics		Income	Level^a^		
	Low (N = 172)	Moderate (N = 324)	Middle (N = 166)	High (N = 68)	*P*-value
Age, mean (SD)	14.0 (3.3)	13.4 (3.4)	13.5 (3.3)	14.3 (3.3)	0.07
Male, No. (%)	82 (47.7)	156 (48.1)	84 (50.6)	22 (32.3)	0.07
Any Medical diagnosis, No. (%)	114 (66.3)	209 (64.5)	125 (75.3)	43 (69.1)	0.10
Any current Medications, No. (%)	67 (38.9)	121 (37.3)	77 (46.4)	38 (55.9)	0.02
BMI percentile, mean (sd)	75.2 (25.9)	77.6 (24.9)	75.9 (27.0)	69.1 (28.3)	0.11
Race^b,c^					
Caucasian, No. (%)	8 (12.7)	16 (25.4)	20 (31.7)	19 (30.2)	
African American, No. (%)	108 (25.3)	203 (47.6)	91 (21.4)	24 (5.6)	
Hispanic, No. (%)	51 (26.1)	88 (45.1)	41 (21.0)	15 (7.7)	
Other/ Unknown, No. (%)	5 (10.9)	17 (37.0)	14 (30.4)	10 (21.7)	
					< .001
Health insurance type^d^					
Private, No. (%)	45 (26.6)	95 (30.3)	75 (46.0)	40 (58.8)	
Public, No. (%)	124 (73.4)	218 (69.7)	87 (54.0)	28 (41.2)	
					< .001

^a^Income level definitions: Low income level- estimated family median income <50 % of national median; Moderate income level- estimated family median income >/=50- <80 % of national median; Middle income level- estimated family median income >/=80-120 % of national median; Upper income level- estimated family median income >120 % of national median. https://www.ffiec.gov/census/htm/2010CensusInfoSheet.htm

^b^Self-identified race/ ethnicity by parent/ guardian or patient as recorded in their electronic medical record

^c^χ^2^_9_ = 62, *p* < .0001 Caucasian and Other tended to be in the Middle and High categories compared with Hispanic and African American

^d^χ^2^_3_ = 37.3 Private insurance was more prevalent in higher income categories

When the studied population was categorized by income level, the groups differed based on race/ethnicity (*p* < .001), frequency of any prescribed medications (*p* = .02) and private health insurance provider (*p* < .001). Caucasian patients came from predominantly middle and high income level neighborhoods (*p* < .001, Table 1) compared to African American and Hispanic patients. Income groups were similar with regard to age, sex, and BMI percentiles.

No relationship was identified between TC level and income level by univariate analysis, or by multivariate analyses adjusting for BMI percentile, age, sex, race/ethnicity and site (BCH vs. BMC), as shown in Table 2.

**Table 2:** Unadjusted and adjusted analysis of mean lipid levels by FFIEC geocoding designated income level

Lipid Levels		Income	Level			
	Low	Moderate	Middle	High	*P*-Value	Adjusted *p*-value^b^
TC mean (sd)	155.2 (26.9)	153.5 (30.4)	155.3 (26.6)	155.5 (27.9)	0.87	0.84
HDL mean (sd)	51.1 (13.3)	47.9 (11.5)	50.2 (13.5)	49.9 (13.2)	0.07	0.08
LDL mean (sd)	89.7 (21.2)	88.7 (29.5)	88.8 (24.4)	87.3 (25.5)	0.97	0.74
TG^a^ mean (sd)	79.0 (41.0)	99.6 (65.8)	92.9 (92.3)	96.4 (49.8)	0.04	0.16

*HDL*, high density lipoprotein, *LDL*, low density lipdoprotein, *TC*, total cholesterol, *TG*, triglycerides

^a^All means are in table are raw means. P-values for triglycerides are from an analysis of log transformed triglyceride scores. Unit for all lipid mean values is mg/dl

^b^Adjusted analysis adjust of BMI percentile, age, sex, race [4] and site [2]

There was an association between TG and income level, but no association between LDL and HDL and income level was demonstrated. Univariate analysis of TG by income levels found significantly lower TG levels in patients of low income level (TG = 79 mg/dl) compared to moderate, middle and high income levels (*p* = .04, Table 2); however, significance was not sustained for either association on multivariate analysis; instead BMI percentile and Caucasian race were identified as significant positive correlates of TG.

## Discussion

In our retrospective review of pediatric patients seen in two urban academic primary care practices we found no relationship between TC, LDL, or HDL and income level by univariate or multivariate analyses. TG levels were lower in patients from low income level compared to all other income levels on univariate analysis but this finding was not sustained on multivariable analysis, likely due to interactions between income level, race/ethnicity and BMI.

Previous studies in adults have shown individual socioeconomic indicators, including income level, to be associated with dyslipidemias [12, 29]. Pediatric studies have also reported relationships between socioeconomic indicators and lipid levels but have been limited by their choice of socioeconomic indicator and diversity in age and ethnicity in the studied cohort. Their results have also been equivocal. Three prospective studies using the following socioeconomic indicators, self-reported education and occupation, school administrator report of school socioeconomic status and the use of free meal vouchers, and maternal education and income adequacy, found no association between these socioeconomic indicators and lipid levels [17, 18, 30]. These studies were limited by restricted age range, lack of ethnic/racial diversity or unvalidated socioeconomic indicators. Two large cohort studies using the NHANES database examined for an association between individual socioeconomic indicators, household income and food security, with cardiovascular risk factors including dyslipidemia [14, 15]. Ali MK et al. identified males between the ages of 6 and 17 from lower income households to have greater prevalence of obesity, and females of older age, 18–24 years of age, from lower income households to have greater prevalence of elevated non-HDL cholesterol levels [14]. Tester JM et al. reported that adolescents, 12–18 years of age, with marginal food security had greater odds of elevated TG levels, than adolescents without food insecurity [15]. These studies were primarily limited by the exclusion of children less than 6 years of age. One prospective study in an adolescent biracial community, reported higher LDL and lower LDL levels in patients with lower parental education [13]. This study was very comprehensive in its metabolic assessment however limited by a narrow age group and limited ethnic/ racial diversity. In our study, we did not identify a relationship between income level and lipid levels. However, the weak relationships seen in prior studies and the lack of studies including the younger pediatric population suggest that additional prospective research is needed to better understand these relationships.

This study had several strengths. Our patient population was diverse with regard to race/ethnicity and the income level based on census tract data. We included the full age range currently eligible for lipid screening, 2 years and older; younger ages were not included in previous studies. The use of two centers improved the generalizability of our findings. The use of census tract data to identify socioeconomic indicators has been previously validated [25–27]. The sample size was adequate to exclude clinically relevant differences in the primary outcome, as suggested by the small difference in mean TC and tight standard deviation between income levels. The presence of higher TG levels in Caucasians seen in our study is consistent with prior studies, suggesting our study cohort was representative of the larger pediatric population [6, 18].

Our study had several limitations. The study cohort had an overall TC lower than reported in nationally representative data from NHANES (154 mg/dl vs. 165 mg/dl), despite higher prevalence of obesity than national rates [1, 6]. The reasons for this are not clear but may reflect temporal trends in lipid levels, or may be a result of other factors known to influence lipid levels such as nutrition, physical activity, and dietary and weight counseling; these behaviors could not be assessed in this retrospective study. We attempted to mitigate any effects of lifestyle counseling by confining our study to subjects with first time cholesterol labs. Similarly, despite national guidelines for cholesterol screening, it is possible that screening practices were biased, resulting in lower cholesterol levels than the general population. In this retrospective study, patients’ fasting state at the moment of the lipid panel lab draw could not be confirmed. A study comparing lipid levels in fasting versus non-fasting children noted minimal, non-clinically significant differences between fasting and non-fasting cholesterol and HDL levels, [31] which are used to calculate non-HDL, one of the lipid screening methodologies recommended by the National Heart, Blood and Lung Institute [32].

## Conclusion

Despite the known association between socioeconomic indicators and lipid levels in adults, income level in childhood was not associated with lipid parameters, including TC, LDL, HDL and TG. A prospective study including self-reported household and individual income level and neighborhood characteristics, including food and park environments, could improve our understanding of these relationships.

## Abbreviations

AAP, American Academy of Pediatrics; BCH, Boston Children’s Hospital; BMC, Boston Medical Center; BMI, body mass index; CVD, cardiovascular disease; EMR, electronic medical record; FFIEC, Federal Financial Institutions Examination Council; HDL, high density lipoprotein; LDL, low density lipoprotein; SES, socioeconomic status; TG- triglycerides

## References

[1] Ogden CL, Carroll MD, Kit BK, Flegal KM (2012). Prevalence of obesity and trends in body mass index among US children and adolescents, 1999–2010. JAMA.

[2] Freedman DS, Dietz WH, Srinivasan SR, Berenson GS (1999). The relation of overweight to cardiovascular risk factors among children and adolescents: the Bogalusa Heart Study. Pediatrics.

[3] Kit BK, Carroll MD, Lacher DA, Sorlie PD, DeJesus JM, Ogden C (2012). Trends in serum lipids among US youths aged 6 to 19 years, 1988–2010. JAMA.

[4] McNiece KL, Poffenbarger TS, Turner JL, Franco KD, Sorof JM, Portman RJ (2007). Prevalence of hypertension and pre-hypertension among adolescents. J Pediatr.

[5] Messiah SE, Arheart KL, Natale RA, Hlaing WM, Lipshultz SE, Miller TL (2012). BMI, waist circumference, and selected cardiovascular disease risk factors among preschool-age children. Obesity.

[6] Hickman TB, Briefel RR, Carroll MD, Rifkind BM, Cleeman JI, Maurer KR (1998). Distributions and trends of serum lipid levels among United States children and adolescents ages 4–19 years: data from the Third National Health and Nutrition Examination Survey. Prev Med.

[7] Can M, Piskin E, Guven B, Acikgoz S, Mungan G (2013). Evaluation of serum lipid levels in children. Pediatr Cardiol.

[8] Berenson GS (2002). Childhood risk factors predict adult risk associated with subclinical cardiovascular disease. The Bogalusa Heart Study. Am J Cardiol.

[9] Berenson GS, Srinivasan SR, Bao W, Newman WP, Tracy RE, Wattigney WA (1998). Association between multiple cardiovascular risk factors and atherosclerosis in children and young adults. The Bogalusa Heart Study. N Engl J Med.

[10] Petkeviciene J, Klumbiene J, Kriaucioniene V, Raskiliene A, Sakyte E, Ceponiene I (2015). Anthropometric measurements in childhood and prediction of cardiovascular risk factors in adulthood: Kaunas cardiovascular risk cohort study. BMC Public Health.

[11] Ferrie JE, Martikainen P, Shipley MJ, Marmot MG (2005). Self-reported economic difficulties and coronary events in men: evidence from the Whitehall II study. Int J Epidemiol.

[12] Chichlowska KL, Rose KM, Diez-Roux AV, Golden SH, McNeill AM, Heiss G (2008). Individual and neighborhood socioeconomic status characteristics and prevalence of metabolic syndrome: the Atherosclerosis Risk in Communities (ARIC) Study. Psychosom Med.

[13] Goodman E, McEwen BS, Huang B, Dolan LM, Adler NE (2005). Social inequalities in biomarkers of cardiovascular risk in adolescence. Psychosom Med.

[14] Ali MK, Bullard KM, Beckles GL, Stevens MR, Barker L, Narayan KM (2011). Household income and cardiovascular disease risks in U.S. children and young adults: analyses from NHANES 1999–2008. Diabetes Care.

[15] Tester JM, Laraia BA, Leung CW, Mietus-Snyder ML (2016). Dyslipidemia and Food Security in Low-Income US Adolescents: National Health and Nutrition Examination Survey, 2003–2010. Prev Chronic Dis.

[16] McGrath JJ, Matthews KA, Brady SS (2006). Individual versus neighborhood socioeconomic status and race as predictors of adolescent ambulatory blood pressure and heart rate. Soc Sci Med.

[17] Resnicow K, Morley-Kotchen J, Wynder E (1989). Plasma cholesterol levels of 6585 children in the United States: results of the know your body screening in five states. Pediatrics.

[18] Frerichs RR, Srinivasan SR, Webber LS, Berenson GR (1976). Serum cholesterol and triglyceride levels in 3,446 children from a biracial community: the Bogalusa Heart Study. Circulation.

[19] Galobardes B, Smith GD, Lynch JW (2006). Systematic review of the influence of childhood socioeconomic circumstances on risk for cardiovascular disease in adulthood. Ann Epidemiol.

[20] US Preventive Services Task Force. Screening for lipid disorders in children: US Preventive Services Task Force recommendation statement. Pediatrics. 2007;120:e215–9. 10.1542/peds.2006-1812 17606545

[21] Kuczmarski RJ, Ogden CL, Grummer-Strawn LM, Flegal KM, Guo SS, Wei R, et al. CDC growth charts: United States. Advance Data. 2000;1–27. 11183293

[22] Kuczmarski RJ, Ogden CL, Guo SS, Grummer-Strawn LM, Flegal KM, Mei Z (2000). CDC Growth Charts for the United States: methods and development. Vital and health statistics Series 11. Data from the National Health Survey.

[23] Federal Financial Institutions Examination Council. FFIEC Geocoding System http://www.ffiec.gov/Geocode/default.aspx Access Years 2009–2011

[24] Federal Financial Institutions Examination Council System. https://www.ffiec.gov/census/htm/2010CensusInfoSheet.htm Access Year 2016

[25] Krieger N (1992). Overcoming the absence of socioeconomic data in medical records: validation and application of a census-based methodology. Am J Public Health.

[26] Krieger N, Chen JT, Waterman PD, Rehkopf DH, Subramanian SV (2005). Painting a truer picture of US socioeconomic and racial/ethnic health inequalities: the Public Health Disparities Geocoding Project. Am J Public Health.

[27] Diez-Roux AV, Kiefe CI, Jacobs DR, Haan M, Jackson SA, Nieto FJ (2001). Area characteristics and individual-level socioeconomic position indicators in three population-based epidemiologic studies. Ann Epidemiol.

[28] Harris PA, Taylor R, Thielke R, Payne J, Gonzalez N, Conde JG (2009). Research electronic data capture (REDCap)--a metadata-driven methodology and workflow process for providing translational research informatics support. J Biomed Inform.

[29] Reppert A, Steiner BF, Chapman-Novakofski K (2008). Prevalence of metabolic syndrome and associated risk factors in Illinois. American Journal of Health Promotion: AJHP.

[30] van den Berg G, van Eijsden M, Vrijkotte TG, Gemke RJ (2012). Socioeconomic inequalities in lipid and glucose metabolism in early childhood in a population-based cohort: the ABCD-Study. BMC Public Health.

[31] Steiner MJ, Skinner AC, Perrin EM (2011). Fasting might not be necessary before lipid screening: a nationally representative cross-sectional study. Pediatrics.

[32] Expert Panel on Integrated Guidelines for Cardiovascular H, Risk Reduction in C, Adolescents, National Heart L, Blood I. Expert panel on integrated guidelines for cardiovascular health and risk reduction in children and adolescents: summary report. Pediatrics. 2011;128(Suppl 5): S213-56. 10.1542/peds.2009-2107C PMC4536582 22084329

